# Attrition and representativeness in development and validation of online symptom checkers—a case study on the *Rheumatic*? Questionnaire

**DOI:** 10.3389/frai.2026.1815241

**Published:** 2026-05-08

**Authors:** Floor Dijkstra Zegers, Ling Qin, Daniyal Selani, Georgy Gomon, Tjardo Maarseveen, Kasper Glas, Astrid van Tubergen, Yvonne Goekoop Ruiterman, Marcel Reinders, Erik van den Akker, Corne Baatenburg de Jong, Lars Klareskog, Barbara Axnäs, Reinhard Bos, Saskia le Cessie, Rachel Knevel

**Affiliations:** 1 Department of Biomedical Data Sciences, Leiden University Medical Center, Leiden, Netherlands; 2 Department of Rheumatology, Leiden University Medical Center, Leiden, Netherlands; 3 Department of Intelligent Systems, Pattern Recognition and Bioinformatics, Delft University of Technology, Delft, Netherlands; 4 Reumazorg Zuid-West Nederland, Roosendaal, Netherlands; 5 Department of Rheumatology, Maastricht University Medical Centre+, Maastricht, Netherlands; 6 Care and Public Health Research Institute (CAPHRI), Maastricht University, Maastricht, Netherlands; 7 Department of Clinical Epidemiology, Haga Hospital, The Hague, Netherlands; 8 Department of Clinical Epidemiology, Leiden University Medical Center, Leiden, Netherlands; 9 Research, Innovation and Impact Funding, ReumaNederland (Dutch Arthritis Society), Amsterdam, Netherlands; 10 Division of Rheumatology, Department of Medicine, Karolinska Institutet, Stockholm, Sweden; 11 Elsa Science, Stockholm, Sweden; 12 Department of Rheumatology, Medical Center Leeuwarden, Leeuwarden, Netherlands; 13 Department of Rheumatology, Nij Smellinghe Hospital, Drachten, Netherlands

**Keywords:** digital decision support system, generalizability, health informatics, musculoskeletal complaints, prediction model, real-world data

## Abstract

**Background:**

Online symptom checkers are often developed and validated on data subject to self-selection and selective attrition, potentially introducing biases in prediction models.

**Objectives:**

To assess recruitment, selection, and attrition patterns in a large Dutch online symptom checker for musculoskeletal complaints and to evaluate potential biases by comparing participant characteristics across recruitment sources and with external target populations.

**Methods:**

Using data from the online Dutch *Rheumatic*? Questionnaire on musculoskeletal complaints, we compared baseline characteristics and key self-reported symptoms between responders to the follow-up survey and nonresponders. The survey responders were furthermore compared according to source of recruitment to the questionnaire, i.e., via primary care clinics, secondary care clinics, or via different online sources. Sex, age and BMI distributions from the total study group were compared to external data of potential target populations of primary and secondary care patients within the Netherlands.

**Results:**

The total study group of answers to the questionnaire comprised 31,457 responders, of which 50% (*n* = 15,591) responded to the follow-up survey. Study participants were predominantly female (76%), middle-aged (one-third 50–60 years), never-smokers (66%), and overweight. While participants recruited through healthcare settings resembled target populations, follow-up survey responders were older, had more rheumatic diagnoses (49% vs. 32%), and reported more symptoms than non-responders. Participant characteristics varied by recruitment source, with social media attracting younger females while healthcare routes reached more diverse populations with varying symptom presentations.

**Conclusion:**

Patterns of recruitment and attrition produced differences in participant characteristics. Healthcare-based recruitment yielded participants resembling intended target populations, and follow-up survey responders differed on some points from nonresponders. Awareness of these selection processes is essential when using real-world symptom checker data for model development.

## Introduction

Online symptom checkers (OSCs) and digital triage tools promise the possibility of optimizing the diagnostic process, as well as aiding health professionals in decision making, therefore aiding the promotion of health equity (Abramoff et al., 2023). For an OSC to be of value, it is crucial that it performs well on the intended target population. Many OSCs rely solely on textbook knowledge or are validated only using clinical vignettes or physician review of patient records (Wallace et al., 2022; El-Osta et al., 2022; Fraser et al., 2018), limiting their validity. Current recommendations promote that OSCs should be developed and validated using real-world data from broadly representative populations (Wallace et al., 2022; Sperrin et al., 2022).

However, using real-world data introduces methodological challenges. The PROBAST (Prediction model Risk Of Bias ASsessment Tool) guidelines describes several domains of risk of bias when using real-world data for model development (Wolff et al., 2019). Particularly, they highlight selective participation and incomplete follow-up. If certain groups such as healthier users or individuals with milder symptoms, are less likely to participate or complete follow-up, systematic bias may arise, leading to reduced generalizability of prediction models to the intended users (Wolff et al., 2019; Lasko et al., 2024; Bethlehem, 2010; Gustavson et al., 2012; de Leeuw and Lugtig, 2015; Keiding and Louis, 2016). Selective dropout may further distort associations between predictors and outcomes, particularly when outcomes depend on follow-up surveys. Therefore, evaluating recruitment sources, participant characteristics, and follow-up completeness is essential to understand potential selection bias when real-world data are used for OSC development.

Although potential problems with selective participation, such as the risk of collider bias, have been discussed (Munafo et al., 2018), few studies so far have described the selection processes in real-world online recruitment for OSCs.

The European SPIDeRR project (Stratification of Patients using advanced Integrative modeling of Data Routinely acquired for diagnosing rheumatic complaints) offers a relevant test case. The project aims to develop an OSC specifically for people with musculoskeletal complaints (MSCs). Musculoskeletal symptoms are highly frequent, comprising 22% of the consultations of primary care physicians in the Netherlands (der van Linden et al., 2004; Finckh, 2009; Raciborski et al., 2017; Jain et al., 2023; Knitza et al., 2021). Despite the benefits of early diagnosis, diagnostic delays remain common (Raciborski et al., 2017), prompting many individuals to seek online explanations for their symptoms (Jain et al., 2023; Knitza et al., 2021; Knevel et al., 2022).

SPIDeRR’s OSC, *Rheumatic*? (Knevel et al., 2022) is a questionnaire collecting information on symptoms, past diagnoses and basic characteristics (Lundberg et al., 2023). The OSC aims to provide accurate disease probabilities for different settings: (1) people online with MSCs, (2) general practitioners (GPs) using the OSC for triage and referral, and (3) for rheumatologists to optimize the diagnostic process. Participants are followed for 1 year to collect information on subsequent referral, diagnosis and medication.

In this study, we assessed recruitment, selection, and attrition patterns among users of the *Rheumatic*? Questionnaire. We compared participant characteristics across recruitment sources and with external data from Dutch primary and secondary care populations with MSCs. In addition, we examined selective follow-up by comparing baseline-only participants with those who completed the first follow-up survey.

## Methods

### Study design and setting

*Rheumatic*? (Elsa Science AB, 2024) is an online questionnaire about current symptoms, past diagnoses and demographic information (all questions provided in Supplementary Table 1). Participants were recruited through multiple channels: online advertisements, the website and newsletters of the Dutch Arthritis Society (ReumaNederland), primary care (GP) clinics, and secondary care rheumatology outpatient clinics. Participants consented to be contacted for follow-up. Four follow-up surveys were sent via e-mail. The first immediately after the baseline questionnaire (included questions about source of recruitment, user-experience and further diagnoses), and the last three after 3, 6 and 12 months, including questions regarding subsequent rheumatologic diagnoses, medication usage, GP visits and referrals.

### Data processing

We included Dutch adults who completed *Rheumatic*? Between 24 July 2021 and 13 July 2023 and provided informed consent. Responses were excluded if demographic information was missing or if email addresses were missing, duplicated, or invalid, ensuring that follow-up questionnaires could be delivered and linked. For the first follow-up survey, only fully completed surveys submitted within 7 days of baseline were included.

### Variables

For the present study we considered age, sex, height, weight, smoking and alcohol habits, rheumatic diagnosis within near family and key symptoms (pain, morning stiffness, all day stiffness, exhaustion, reduced endurance or none). Age was classified according to <40, 40–50, 50–60 and 60+ years. Body mass index (BMI) was derived from midpoint values of weight and height categories (5 kg and 5 cm intervals) and grouped as <18.5, 18–24.9, 25–30 and 30+ (Weir and Jan, 2024). Family diagnosis was defined as reporting a rheumatic-related diagnosis within close family (yes/no/do not know). Previous diagnosis was defined as reporting any previously given rheumatic-related diagnosis at baseline or in the follow-up survey (yes/no/do not know). Recruitment sources (via a rheumatology clinic, GP, the ReumaNederland website/newsletters, other online sources (with a free text option) or other sources) and further rheumatic diagnoses were solely indicated in the follow-up survey.

### Website analytics

Daily inclusion counts were compared with daily numbers of Dutch new visitors to the website (Elsa Science AB, 2024) hosting the questionnaire between July 2021 and December 2023. All website analytics were summarized using Google Analytics (Google Analytics, 2024).

### Expected target populations

To assess representativeness, we collected data from the same time-period (24 July 2021 to 13 July 2023) from (i) all GP episodes with MSCs-related ICPC-codes (L01–L20, L29, L83–L94, L98, L99, T92, thus excluding MSC consultations with fractures, infections and innate diseases) (Hofmans-Okkes and Lamberts, 1996; Ardesch et al., 2023), (ii) regional rheumatology clinics in the South West Netherlands (RZWN), and (iii) the academic rheumatology clinic of Leiden University Medical Centre (LUMC). Age, sex and BMI (when available) were compared between the study population and these potential target populations.

### Statistical analysis

Contingency tables summarized baseline characteristics of all participants and compared responders vs. nonresponders to the first follow-up survey. Differences in percentages were presented with 95% confidence intervals (CIs) and visualized in a forest plot.

Recruitment source differences were assessed using contingency tables stratified by reported source of recruitment coded as GP, rheumatology clinic, other sources in general, and online sources. The latter was subdivided based on free text answers into the ReumaNederland website, Facebook, Google, Instagram, or other online sources. We used only participants’ answers to the first follow-up survey since this survey contained information on sources of recruitment.

Representativeness relative to target populations was assessed by comparing distributions of age, sex, and BMI between responders to the follow-up survey and external clinical populations, grouped as above. In the external data, BMI was only calculated when available.

Recruitment patterns over time were examined using frequency plots of inclusion date and Google hit-data with 7-day rolling means and furthermore stratified by recruitment source for responders to the follow-up survey.

All data handling and analysis were performed using R version 4.1.3 (R Core Team, 2023), using ggplot2 for plots (Wickham, 2016), and gt and gtsummary for tables (Sjoberg et al., 2021; Iannone et al., 2024).

## Results

### Characteristics of all *Rheumatic*? Participants

After data processing 31,159 participants were included (Supplementary Figure 1). Participants were predominantly female (76%), most commonly aged 50 to 60 years (31%), never-smokers (66%) and frequently overweight. About half (51%) reported no previous rheumatic diagnosis, and 48% reported a close family member having a rheumatic diagnosis. Pain (83%) and morning stiffness (62%) were the most frequent symptoms. 29% reported swelling in one or more body parts and 1% reported having none of the key symptoms. Characteristics of participants are fully specified in Supplementary Table 2.

### Differences between follow-up survey participants and non-participants

**Figure 1:**
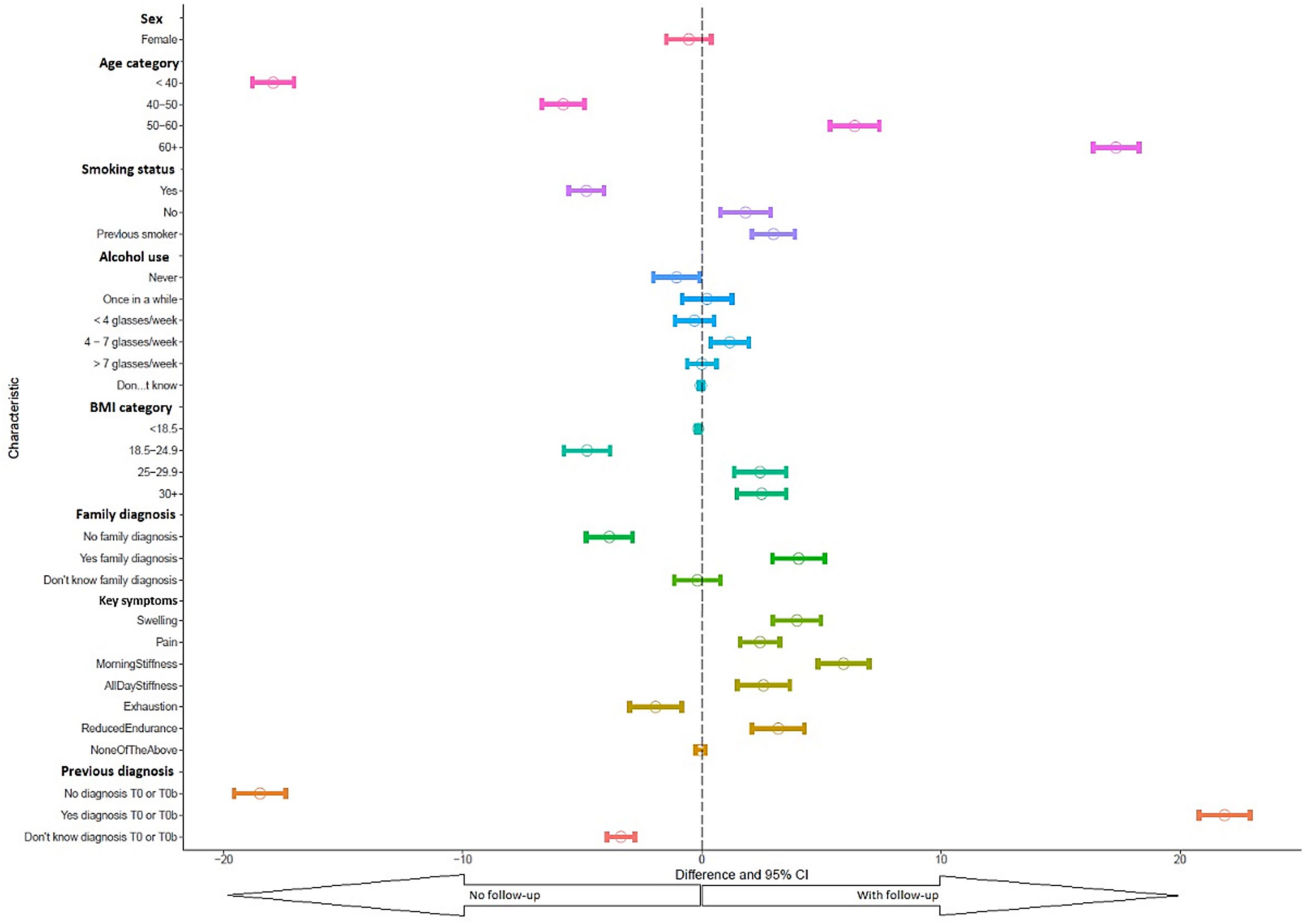
Forest plot of difference in characteristics between responders and nonresponders in percentage.

Of all participants, half (*N* = 15,591) responded to the first follow-up survey. Characteristics of follow-up responders and nonresponders are presented in Supplementary Table 2, and differences in percentage are shown in Figure 1 and Supplementary Table 2. Follow-up participation did not yield differences in sex, alcohol consumption, family history of rheumatic disease, or having a complete absence of key symptoms. However, responders were older, more frequently reported a prior rheumatic diagnosis (49% vs. 32%), were less frequently current smokers and more often had a BMI above 25. Responders more frequently reported key symptoms, except for exhaustion which was reported more frequently among nonresponders than responders.

### Characteristics by source of recruitment

Most of the participants with information on recruitment [12,574 (81%)] were recruited online, with the largest proportion originating from ReumaNederland communication channels, Google, and social media platforms (Table 1). Smaller proportions were recruited through secondary care rheumatology clinics, general practitioners (GPs), or other offline sources.

**Figure 2:**
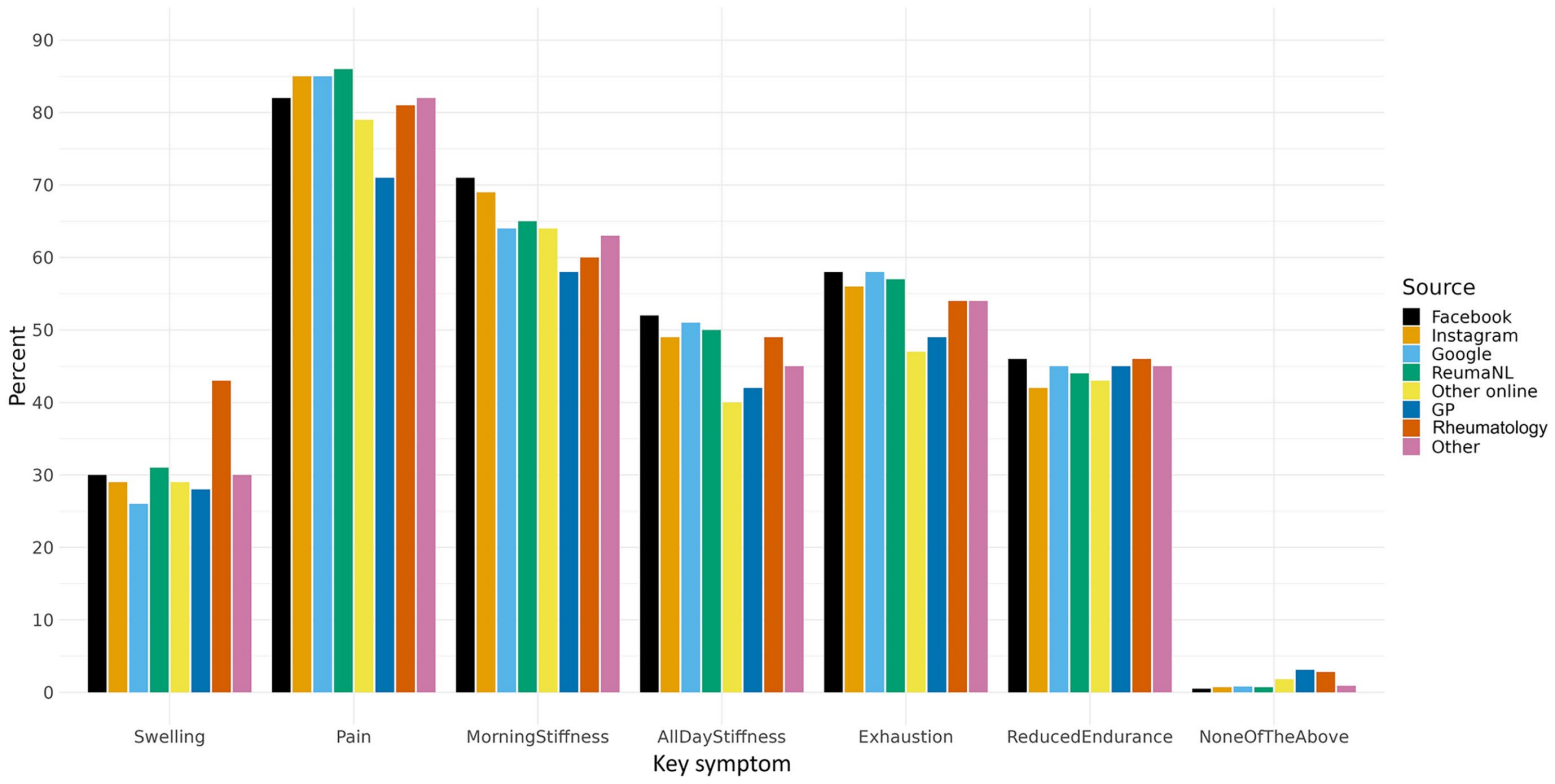
Barplot of reported key symptoms according to recruitment source.

*Social media recruitment* predominantly reached younger women, with women accounting for 95 to 98% of participants recruited via Facebook and Instagram. These participants also showed slightly higher BMI values, with the highest prevalence of obesity observed among Facebook recruits.
*Other online recruitment* sources, including Google and disease-specific websites, reached a more heterogeneous population. Participants recruited via Google less often had a prior rheumatic diagnosis.
*Healthcare-based recruitment* through GPs and rheumatology clinics resulted in more balanced sex distributions and older age profiles. Participants recruited via rheumatology clinics most frequently reported swelling, while those recruited through GPs reported fewer symptoms overall (Figure 2).

**Table 1:** Characteristics according to source of recruitment for participants responding to the follow-up survey.

Characteristic	Facebook, *N* = 929 (6.0%)	Instagram, *N* = 289 (1.9%)	Google, *N* = 1,266 (8.1%)	ReumaNL^b^, *N* = 9,589 (62%)	Other online, *N* = 501 (3.2%)	GP, *N* = 98 (0.6%)	Rheumatology, *N* = 1,267 (8.1%)	Other, *N* = 1,652 (11%)
Sex								
Female	881 (95%)	282 (98%)	933 (74%)	7,181 (75%)	396 (79%)	54 (55%)	813 (64%)	1,304 (79%)
Age category								
<40	59 (6.4%)	45 (16%)	255 (20%)	1,184 (12%)	32 (6.4%)	13 (13%)	134 (11%)	141 (8.5%)
40–50	200 (22%)	65 (22%)	290 (23%)	1,731 (18%)	48 (9.6%)	9 (9.2%)	172 (14%)	238 (14%)
50–60	541 (58%)	139 (48%)	380 (30%)	3,162 (33%)	160 (32%)	23 (23%)	378 (30%)	591 (36%)
60+	129 (14%)	40 (14%)	341 (27%)	3,512 (37%)	261 (52%)	53 (54%)	583 (46%)	682 (41%)
Smoking status								
Yes	103 (11%)	19 (6.6%)	157 (12%)	956 (10.0%)	37 (7.4%)	14 (14%)	175 (14%)	175 (11%)
No	621 (67%)	217 (75%)	808 (64%)	6,488 (68%)	343 (68%)	62 (63%)	791 (62%)	1,084 (66%)
Previous smoker	205 (22%)	53 (18%)	301 (24%)	2,145 (22%)	121 (24%)	22 (22%)	301 (24%)	393 (24%)
Alcohol use								
Never	245 (26%)	74 (26%)	294 (23%)	2,365 (25%)	120 (24%)	19 (19%)	331 (26%)	430 (26%)
Once in a while	376 (40%)	113 (39%)	458 (36%)	3,173 (33%)	143 (29%)	41 (42%)	414 (33%)	602 (36%)
<4 glasses/week	137 (15%)	55 (19%)	201 (16%)	1,604 (17%)	98 (20%)	13 (13%)	193 (15%)	249 (15%)
4–7 glasses/week	114 (12%)	41 (14%)	186 (15%)	1,604 (17%)	86 (17%)	17 (17%)	233 (18%)	241 (15%)
>7 glasses/week	56 (6.0%)	6 (2.1%)	124 (9.8%)	833 (8.7%)	53 (11%)	7 (7.1%)	89 (7.0%)	126 (7.6%)
Do not know	1 (0.1%)	0 (0%)	3 (0.2%)	10 (0.1%)	1 (0.2%)	1 (1.0%)	7 (0.6%)	4 (0.2%)
BMI category								
<18.5	0 (0%)	1 (0.3%)	1 (<0.1%)	4 (<0.1%)	0 (0%)	0 (0%)	0 (0%)	2 (0.1%)
18.5–24.9	130 (14%)	66 (23%)	349 (28%)	2,343 (24%)	122 (24%)	22 (22%)	273 (22%)	356 (22%)
25–29.9	396 (43%)	124 (43%)	509 (40%)	4,142 (43%)	224 (45%)	47 (48%)	541 (43%)	696 (42%)
30+	403 (43%)	98 (34%)	407 (32%)	3,100 (32%)	155 (31%)	29 (30%)	453 (36%)	598 (36%)
Family diagnosis								
No	250 (27%)	75 (26%)	338 (27%)	2,351 (25%)	113 (23%)	21 (21%)	314 (25%)	375 (23%)
Yes	486 (52%)	166 (57%)	587 (46%)	4,826 (50%)	273 (54%)	45 (46%)	625 (49%)	843 (51%)
Do not know	193 (21%)	48 (17%)	341 (27%)	2,412 (25%)	115 (23%)	32 (33%)	328 (26%)	434 (26%)
Previous diagnosis^a^								
No	367 (40%)	137 (47%)	694 (55%)	3,900 (41%)	175 (35%)	34 (35%)	395 (31%)	523 (32%)
Yes	528 (57%)	138 (48%)	474 (37%)	5,174 (54%)	301 (60%)	55 (56%)	764 (60%)	1,031 (62%)
Do not know	34 (3.7%)	14 (4.8%)	98 (7.7%)	515 (5.4%)	25 (5.0%)	9 (9.2%)	108 (8.5%)	98 (5.9%)

a Self-reported rheumatic-related diagnosis at baseline or in the follow-up survey.

b ReumaNL, ReumaNederland.

Family history of rheumatic disease varied modestly across recruitment sources, and smoking patterns showed little variation.

### Recruitment tendencies

**Figure 3:**
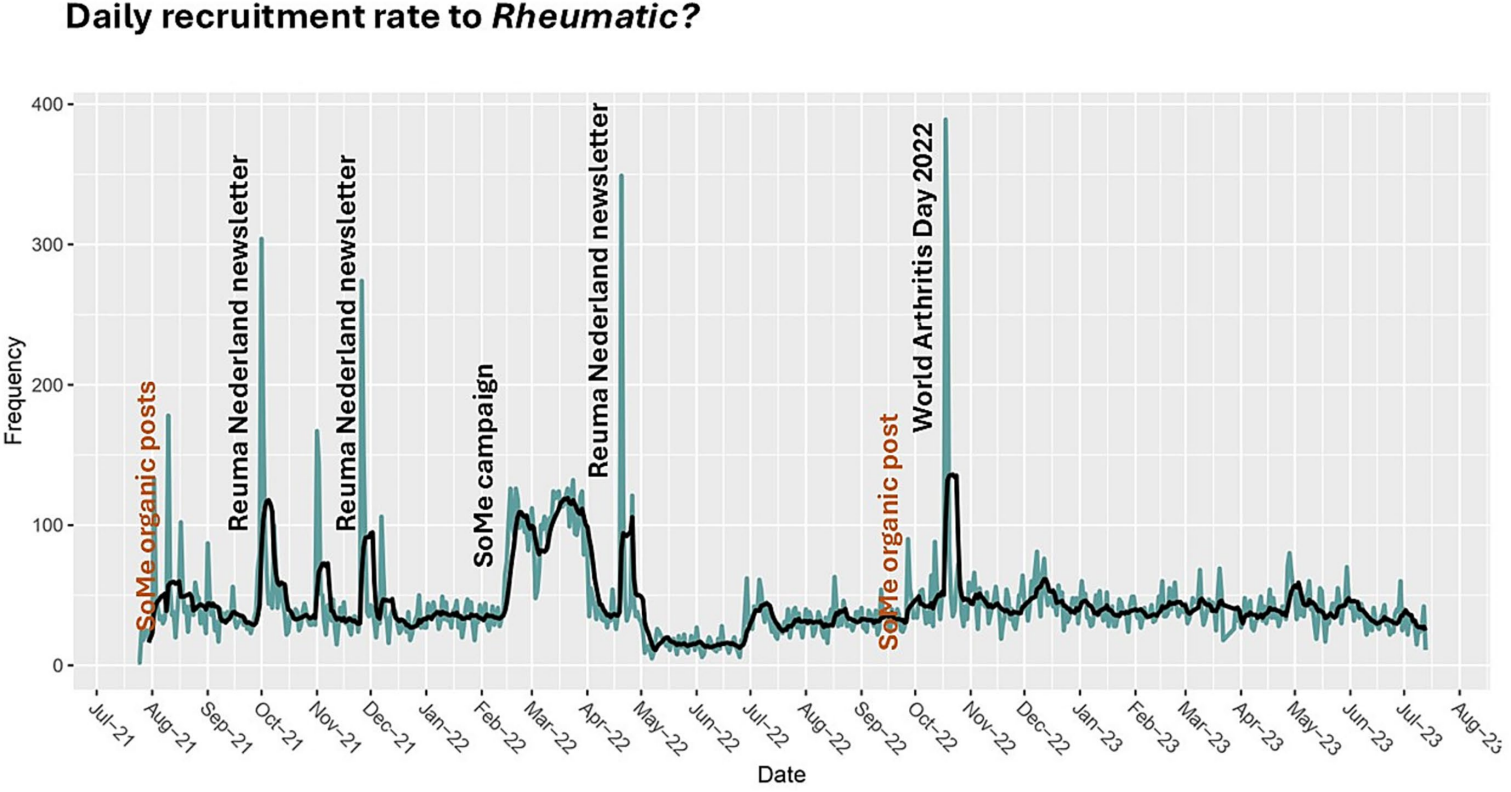
Daily recruitment rate in the study.

Daily recruitment rates varied over time and are shown in Figure 3. Distinct recruitment peaks coincided with newsletter campaigns conducted by ReumaNederland, annual World Arthritis Day and targeted social media campaigns. Source-stratified analyses highlighted the effect of paid social media campaigns on increases in recruitment, whereas organic posts on the ReumaNederland social media site had limited impact (Supplementary Figure 2). Daily recruitment closely followed traffic to the questionnaire website (Supplementary Figure 3).

### Comparison with target populations in primary and secondary care

Compared with patients from Dutch secondary care rheumatology clinics, the overall *Rheumatic*? Cohort included a higher proportion of women. However, participants recruited via secondary care closely resembled secondary care patients with respect to sex. Patients from the academic rheumatology clinic were younger and were more present in the lower BMI groups. However, participants from the regional rheumatology clinic resembled *Rheumatic*? Rheumatology recruits more closely (Table 2).

**Table 2:** Comparison of the participants responding to the follow-up survey with external data.

	*Rheumatic?* Questionnaire			External	External	
				Primary care	Rheumatology clinics	
Reported characteristic *n* (%)	All *N* = 15,591	Recruited via GP, *N* = 98	Recruited via rheumatology clinics, *N* = 1,267	ELAN^a^^,^^b^, *N* = 303,245	RZWN^a^^,^^c^, *N* = 14,195	LUMC^a^^,^^d^, *N* = 4,040
Sex						
Female	11,844 (76%)	54 (55%)	813 (64%)	177,077 (59%)	9,567 (67%)	2,623 (65%)
Missing	0	0	0	4,540	0	0
Age						
<40	1,863 (12%)	13 (13%)	134 (11%)	52,238 (18%)	2,693 (19%)	992 (25%)
40–50	2,753 (18%)	9 (9.2%)	172 (14%)	52,957 (18%)	2,333 (16%)	691 (17%)
50–60	5,374 (34%)	23 (23%)	378 (30%)	67,071 (22%)	3,457 (24%)	888 (22%)
60+	5,601 (36%)	53 (54%)	583 (46%)	126,155 (42%)	5,712 (40%)	1,469 (36%)
Missing	0	0	0	4,824	0	0
BMI						
<18.5	35 (0.2%)	0 (0%)	2 (0.2%)	1,590 (1%)	188 (1.5%)	26 (2%)
18.5–24.9	3,771 (24%)	24 (24%)	285 (22%)	43,625 (25%)	4,155 (33%)	465 (40%)
25–29.9	6,542 (42%)	45 (46%)	527 (42%)	70,727 (40%)	4,048 (32%)	394 (34%)
30+	5,243 (34%)	29 (30%)	453 (36%)	59,608 (34%)	4,290 (34%)	277 (24%)
Missing	0	0	0	127,695	1,514	2,878

a Percentages calculated among non-missing.

b ELAN, Extramural LUMC Academic Network.

c RZWN, Reumazorg Zuid West Nederland.

d LUMC, Leiden University Medical Center.

In comparison with primary care data from GPs, women were overrepresented in the overall *Rheumatic*? Cohort. This imbalance was not observed among participants recruited through GPs, whose sex distribution closely matched that of the primary care population. The overall *Rheumatic*? Cohort held relatively more middle-aged participants (50–60 years), and those recruited via GPs tended to be older than individuals in the external GP data. BMI distributions were comparable between all groups (Table 2).

## Discussion

In this cohort study of more than 31,000 individuals with musculoskeletal symptoms using the *Rheumatic*? Online symptom checker (OSC), we identified distinct recruitment, selection, and attrition patterns with direct implications for representativeness and potential bias in real-world OSC data. Recruitment through primary and secondary care settings yielded participant characteristics closely resembling their intended target populations, whereas online recruitment—particularly via social media—resulted in younger and predominantly female participants. In addition, follow-up survey responders differed systematically from nonresponders, indicating systematic attrition.

Our findings are consistent with prior studies of non–disease-specific OSCs, which reported a predominance of female users (62–85%) and younger mean ages (Kopka et al., 2023; Winn et al., 2019; Arellano Carmona et al., 2022; Morse et al., 2020). Pain was also the most frequently reported symptom in those studies, mirroring our observations. Studies on OSCs specifically targeting musculoskeletal complaints remains limited. A small German study evaluating musculoskeletal-focused OSCs and an Australian primary care study reported sex and age distributions broadly comparable to our findings, although sample sizes were substantially smaller (Knitza et al., 2021; Haas et al., 2023). Together, these data suggest that online symptom checkers for musculoskeletal complaints consistently attract selective user groups, particularly when participants are recruited outside healthcare settings.

### Recruitment source and representativeness

Participants recruited through healthcare settings closely matched external primary and secondary care populations with respect to sex, age, and BMI. This alignment suggests that OSC deployment within clinical pathways may yield datasets representative of these intended users. In contrast, participants recruited online were younger and more frequently female, with notable variation between online recruitment channels. Social media based recruitment resulted in an almost exclusively female population, whereas Google-based recruitment reached a more heterogeneous group with fewer prior diagnoses. Importantly, our findings demonstrate that “online users” do not constitute a homogeneous population. Marked differences in demographic and clinical characteristics were observed across online recruitment sources. As other authors previously have suggested, these patterns likely reflect differences in platform use, content targeting, and health-seeking behavior, underscoring that recruitment strategy itself is a key determinant of dataset composition (Bol et al., 2020).

### Selective attrition and follow-up

Follow-up survey responders differed from nonresponders in age, symptom burden, smoking status, and prevalence of prior rheumatic diagnoses. A follow-up response rate of 50% is comparable to other longitudinal studies, including surveys in rheumatology and other domains (Garcia et al., 2014; Wu et al., 2022). The higher rate of follow-up participation among older participants and nonsmokers aligns with previous population-based studies (Okpara et al., 2023; Canivet et al., 2021; Bamer et al., 2020). However, the greater symptom burden and higher prevalence of prior diagnoses among responders suggest that disease severity and health awareness influenced continued participation.

### Implications for prediction model development

Selective attrition complicates the interpretation and generalizability of analyses relying on follow-up data. If associations between predictors and outcomes differ between responders and nonresponders, prediction models derived from follow-up data may be biased. Multiple imputation and sensitivity analyses under alternative missingness mechanisms may be considered to assess robustness (Sterne et al., 2009; van Buuren, 2018). Where feasible, linkage to external data sources may help validate outcomes and improve handling of missing data. In the *Rheumatic*? Study, planned linkage with electronic health records from primary and secondary care may address these limitations.

Before developing prediction models using OSC data, the intended use and target population should be clearly defined. For *Rheumatic*?, potential applications range from a general online self-assessment tool to decision support in primary or secondary care. Each use case implies a different target population and, consequently, different requirements for representativeness.

When discrepancies between the study population and target population exist, statistical approaches such as reweighting or poststratification may improve transportability (Wolff et al., 2019; Keiding and Louis, 2016; Petersen et al., 2024; Zhang et al., 2014). Additionally, when recruitment sources are strongly associated with participant characteristics, incorporating recruitment source as a predictor or exploring interactions with other predictors may be warranted. Separate validation of prediction models across recruitment sources may further enhance robustness. More research is needed to compare different weighting and calibration methods in the case of making prediction models with multiple target groups from a heterogeneous population.

### Handling missing follow-up data

Missing follow-up data introduces additional methodological challenges. When missingness is associated with participant characteristics, standard assumptions such as missing at random must be carefully evaluated. Multiple imputation and sensitivity analyses under alternative missingness mechanisms may be considered to assess robustness (Sterne et al., 2009; van Buuren, 2018; Biering et al., 2015). Where feasible, linkage to external data sources may help validate outcomes and improve handling of missing data. In the *Rheumatic*? Study, planned linkage with electronic health records from primary and secondary care may address these limitations.

### Strengths and limitations

This study provides one of the first large-scale, empirical descriptions of recruitment and participation patterns in an OSC targeting musculoskeletal complaints. The large sample size enabled detailed stratified analyses across recruitment sources and follow-up status. Although formal external reference data for the purely online target population were unavailable, the observed recruitment and attrition patterns reflect well-described mechanisms of self-selection and differential participation in digital health research. These mechanisms are unlikely to be specific to musculoskeletal complaints and may be relevant to a broad range of online symptom checkers and digital triage tools. This study is limited by incompleteness of external reference data: BMI was incomplete, and we lacked measures of socioeconomic status and digital literacy which could have provided extra insight in the comparison. Furthermore, recruitments peaks tied to specific campaigns may have altered the sample composition, limiting inferences about routine use of the symptom checker.

## Conclusion

Our study underlines the challenges that arise when recruiting real-world participants online. Recruitment and attrition processes play a central role in shaping the composition of real-world online symptom checker datasets. In this study, we observed that patterns of recruitment and attrition created differences in participant characteristics. However, we also observed that recruitment via general practitioners and rheumatology clinics yielded participant samples resembling their intended clinical target populations. These patterns reflect general mechanisms of self-selection and differential participation in digital health research and underscore the importance of explicitly evaluating recruitment and attrition when developing and validating prediction models using real-world OSC data.

## Data Availability

The raw data supporting the conclusions of this article will be made available by the authors, without undue reservation. All scripts used are publicly available at: https://github.com/fdzegers/Selection_and_attrition.
