# Supplementary material for: Attrition and representativeness in development and validation of online symptom checkers—a case study on the Rheumatic? Questionnaire
Source: Front Artif Intell. 2026 May 8;9:1815241. doi: 10.3389/frai.2026.1815241 (PMC13194141; doi:10.3389/frai.2026.1815241)

List of tables and figures

eTable 1: All questions included in the *Rheumatic?* questionnaire

eFigure 1: Flowchart

eTable 2: Characteristics of responders and non-responders and difference in characteristics in percent (with 95% CI)

eFigure 2: Source specific inclusion frequencies

eFigure 3: Frequencies of new daily users of the hosting website

eTable 1: Questions included in Rheumatic? baseline questionnaire

Question	**Options**
**Select the options that describe your problems**	Pain/Ache<br>Joint swelling<br>Morning stiffness<br>Stiffness at any time of the day<br>Tiredness or fatigue<br>Reduced endurance<br>None of the above
Pain	
**Which of these alternatives best describes your pain?**	It is tender<br>It is diffuse/dull<br>It is sharp/stabbing<br>It radiates from a certain point<br>It is clearly connected to one or several body parts<br>It moves around<br>None of the above<br>I don't know
**When do you experience pain?**	It is more or less constant and remains during the major part of the day and night<br>It comes and goes<br>It is at its worst in the morning<br>It is at its worst in the evening<br>It is at its worst at night<br>None of the above<br>I don't know
**What cause your pain?**	It is made worse by physical exertion<br>It is made better by physical exertion<br>It occurs when I put pressure on a certain point<br>Sitting or being still makes it worse<br>When initialising movement after rest<br>None of the above<br>I don't know
**How much pain have you experienced during the last week?**	No pain<br>A little pain<br>Pain<br>Much pain<br>Very much pain<br>Worst imaginable pain
**What is the worst level of pain you have experienced due to your problems?**	No pain<br>A little pain<br>Pain<br>Much pain<br>Very much pain<br>Worst imaginable pain
**Where do you usually experience pain?**	Right foot<br>Left foot<br>Right hand<br>Left hand<br>Right leg and hip<br>Left leg and hip<br>Right arm<br>Left arm<br>Head/neck<br>Chest<br>Stomach<br>Back
**Indicate where in your left hand you usually experience pain**	Thumb<br>Base of thumb<br>Top finger joints<br>Middle finger joints<br>Knuckles<br>Wrist<br>The whole hand<br>None of the above
**Indicate where in your right hand you usually experience pain**	Thumb<br>Base of thumb

	Top finger joints<br>Middle finger joints<br>Knuckles<br>Wrist<br>The whole hand<br>None of the above
**Indicate where in your left arm you usually experience pain**	Tendon elbow<br>Elbow<br>Forearm<br>Upper arm<br>Shoulder<br>None of the above
**Indicate where in your right arm you usually experience pain**	Shoulder<br>Upper arm<br>Elbow<br>Tendon elbow<br>Forearm<br>None of the above
**Indicate where in your left foot you usually experience pain**	Ankle<br>Achilles' tendon<br>Big toe<br>Big toe base joint<br>Base joints toes<br>Top toe joints<br>Sole<br>The whole foot<br>None of the above
**Indicate where in your right foot you usually experience pain**	Ankle<br>Achilles' tendon<br>Big toe<br>Big toe base joint<br>Base joints toes<br>Top toe joints<br>Sole<br>The whole foot<br>None of the above
**Indicate where in your left leg you usually experience pain**	Edge of hip<br>Thigh<br>Knee<br>Shin/calf<br>Hip joint<br>None of the above
**Indicate where in your right leg you usually experience pain**	Edge of hip<br>Thigh<br>Knee<br>Shin/calf<br>Hip joint<br>None of the above
**Indicate where in or around your back you usually experience pain**	Upper back<br>Neck<br>Lower back<br>Left buttock<br>Right buttock<br>None of the above
**Have you experienced pain that shifts between your right and left buttock?**	Yes<br>No<br>I don't know
**Do you wake at night because of pain?**	Yes<br>No<br>I don't know
**Does any of the painkillers naproxen, ibuprofen or diclofenac relieve your pain substantially and quickly?**	Yes<br>No<br>I don't know
**Have you got any of the following symptoms?**	Difficulty in controlling your bladder and/or bowels<br>Numbness or reduced sensation around your anus or genitals<br>Numbness, tinglings or weakness in your legs or arms<br>No, I don't have any of these symptoms<br>I don't know
Swelling	
**Indicate where you have swelling or have recently had swelling**	Right foot

	Left foot<br>Right hand<br>Left hand<br>Right leg<br>Left leg<br>Right arm<br>Left arm<br>Head/neck<br>Chest<br>Stomach<br>Back<br>None of the above
**In which way is or has your left hand been swollen?**	The whole hand is swollen<br>One or multiple joints is swollen<br>The palm of my hand is swollen<br>The back of my hand is swollen<br>Whole fingers are swollen<br>None of the above<br>I don't know
**Indicate where your left hand is or has been swollen**	Thumb<br>Base of thumb<br>Top finger joints<br>Middle finger joints<br>Knuckles<br>Wrist<br>None of the above
**In which way is or has your right hand been swollen?**	The whole hand is swollen<br>One or multiple joints is swollen<br>The palm of my hand is swollen<br>The back of my hand is swollen<br>Whole fingers are swollen<br>None of the above<br>I don't know
**Indicate where your right hand is or has been swollen.**	Thumb<br>Base of thumb<br>Top finger joints<br>Middle finger joints<br>Knuckles<br>Wrist<br>None of the above
**Indicate where your left foot is or has been swollen.**	Ankle<br>Achilles' tendon<br>Big toe<br>Big toe base joint<br>Base joints toes<br>Top toe joints<br>Sole<br>The whole foot<br>None of the above
**Indicate where your right foot is or has been swollen.**	Ankle<br>Achilles' tendon<br>Big toe<br>Big toe base joint<br>Base joints toes<br>Top toe joints<br>Sole<br>The whole foot<br>None of the above
**Indicate where your left arm is or has been swollen.**	Elbow<br>Armpit<br>Forearm<br>Upper arm<br>Shoulder<br>None of the above
**Indicate where your right arm is or has been swollen.**	Elbow<br>Armpit<br>Forearm<br>Upper arm<br>Shoulder<br>None of the above

**Indicate where your left leg is or has been swollen.**	Edge of hip<br>Thigh<br>Knee<br>Shin/calf<br>Hip joint<br>None of the above
**Indicate where your right leg is or has been swollen.**	Edge of hip<br>Thigh<br>Knee<br>Shin/calf<br>Hip joint<br>None of the above
**Indicate where on your face or throat you are or have been swollen.**	Right cheek<br>Left cheek<br>Right eye<br>Left eye<br>Right side of the throat<br>Left side of the throat<br>None of the above
General condition	
**Are your joints stiff after inactivity, but feel less stiff after a couple of minutes of movement?**	Yes<br>No<br>I don't know
**Approximately how long does your morning stiffness last?**	0-20 minutes<br>20-30 minutes<br>30-45 minutes<br>45-60 minutes<br>More than 60 minutes
**In which way do you experience tiredness?**	Resting helps<br>Resting does not help, I am still tired<br>Body feels tired<br>Hard to think clearly<br>None of the above<br>I don't know
**How tired have you felt during the past week?**	0 %<br>0–20%<br>20–40%<br>40–60%<br>60–80%<br>80–100%
**Can you relate to any of these descriptions?**	Recurring fever without clear cause<br>Unaccountable weight loss<br>None of the above
**Does your body react differently to physical activity compared to how you are used to it reacting previously?**	I have less stamina than I'm used to<br>I'm not as physically fit as I'm used to<br>I get stiff more easily after exercise than I used to<br>No, I haven't noticed any difference<br>None of the above<br>I don't know
**Have you experienced any of the following problems with your breathing?**	I quickly get out of breath<br>I can only take short breaths<br>I get chest pains when taking deep breaths in<br>I have a dry cough<br>No, I have not experienced any of these problems<br>I don't know
Skin	
**Have you experienced any of the following problems for example with cold, humidity, or stress?**	Fingers becoming whiter or blue<br>Toes becoming whiter or blue<br>Fingers or toes becoming numb<br>No, I have not experienced any of these problems<br>I don't know
**Does it look something like this?**	Yes<br>No<br>I don't know
**Are several fingers or toes usually affected at the same time?**	Yes<br>No<br>I don't know
**How often do you experience this?**	Sometimes, for example when it's cold<br>At least once a month<br>Several times a month, often several days on end

	Daily<br>I don't know
**When did you start experiencing these problems with your fingers or toes?**	During childhood<br>In my teens<br>After 20 years of age<br>After 30 years of age<br>I don't know
**Have you experienced any of these skin conditions during the last year?**	Red and itchy rash after sun exposure<br>Other rashes<br>Skin itches<br>Sores/cracks on fingertips or toes<br>Unaccountable pigmentation changes<br>Other skin problem<br>No, I have not experienced any of these problems<br>I don't know
**Does it take long for the sores/cracks on your fingertips or toes to heal?**	Yes, it often takes more than two weeks<br>It usually heals within two weeks<br>I don't know
**Where on your skin have you had problems?**	Hands<br>Feet<br>Back<br>Chest<br>Buttocks<br>Scalp<br>Legs<br>Arms<br>Face<br>None of the above
**Where on your hand/hands have you experienced these problems?**	The upper side/back of my hand<br>The palm of my hand<br>Fingers<br>Nails or cuticles<br>None of the above
**Where on your face have you experienced these problems?**	Eyelids<br>Across cheeks and bridge of nose, known as butterfly rash<br>None of the above
Other complaints	
**Do you repeatedly experience one or more of these stomach complaints?**	Acid reflux<br>Heartburn<br>Diarrhoea<br>Constipation<br>Difficulty swallowing or it goes down wrong<br>Bloated stomach<br>No, I have not experienced any of these problems<br>I don't know
**Have you experienced one or more of the following problems?**	Difficulty speaking or singing<br>Difficulty opening mouth wide<br>Dry mouth<br>Sores in mouth or nose<br>Dry eyes<br>Hair loss<br>No, I have not experienced any of these problems<br>I don't know
**Have you experienced a continuous state of dry mouth for more than 3 months?**	Yes<br>No<br>I don't know
**Do you need to drink in order to swallow food?**	Yes<br>No<br>I don't know
**Have you experienced a continuous state of dry eyes for more than 3 months?**	Yes<br>No<br>I don't know
**Are you taking any medication that could cause dry eyes or dry mouth?**	Yes<br>No<br>I don't know
**I find it difficult to …**	Open jars<br>Lift a glass and drink from it<br>Hold something, like a paper or credit card, without dropping it<br>Take something down from a shelf at head height<br>Take off a sweater

	Wash my hair<br>Pick up a pen from the floor<br>None of the above
**I find it difficult to …**	Look over my shoulder without turning my body<br>Sit down for more than an hour<br>Stand up for more than an hour<br>Get up from a chair without using my hands<br>Perform light physical activity, like hoovering a room<br>Walk up more than one flight of stairs without resting<br>Walk more than 1 km without resting<br>Walk without falling and stumbling<br>Walk barefoot indoors because it hurts too much<br>None of the above
**Limited flexibility in my joints makes it difficult to …**	Form a fist<br>Open my hand and extend my fingers<br>Bend the wrist<br>Flex neck forward and backward<br>Bend arm and elbow<br>Open arm and straighten elbow<br>Lift arm<br>Lift and rotate arm forwards<br>None of the above<br>I don't know
**Limited flexibility in my joints makes it difficult to …**	Bend my knee<br>Straighten out my knee<br>Lift my leg<br>Lift and rotate leg<br>Flex my ankle<br>Straighten out my ankle<br>None of the above<br>I don't know
**Do you currently have fever?**	Yes<br>No<br>I don't know
Background	
**Approximately how long ago did your problems with pain, swelling or morning stiffness begin?**	Less than 6 weeks ago<br>Between 6 weeks to a year ago<br>1 to 3 years ago<br>3 to 5 years ago<br>More than 5 years ago<br>I don't know
**How old are you?**	Under 20 years<br>20–29 years<br>30–39 years<br>40–49 years<br>50–59 years<br>60–69 years<br>70–79 years<br>Over 80 years
**What is your biological sex?**	Female<br>Male
**How tall are you?**	Under 150 cm<br>150–155 cm<br>155–160 cm<br>160–165 cm<br>165–170 cm<br>170–175 cm<br>175–180 cm<br>180–185 cm<br>185–190 cm<br>190–195 cm<br>195–200 cm<br>Over 200 cm
**How much do you weigh?**	Below 50 kg<br>50-55 kg<br>55 – 60 kg<br>60 – 65 kg<br>65 – 70 kg<br>70 – 75 kg<br>75 –80 kg

	80 – 85 kg<br>85 – 90 kg<br>90 – 100 kg<br>100 – 110 kg<br>Over 110 kg
**Have you experienced any previous trauma in any of the affected joints?**	Sport injury<br>Surgery<br>Other trauma<br>I don't know<br>No, I have not had any previous joint trauma
**Have you ever been diagnosed by a physician with any of the following diseases?**	Rheumatoid arthritis<br>Sjogren's syndrome<br>Systemic lupus erythematosus (SLE)<br>Myositis<br>Systemic sclerosis<br>Axial spondyloarthritis<br>Osteoarthritis<br>Gout<br>Polymyalgia rheumatica<br>Psoriatic arthritis<br>Fibromyalgia<br>Other rheumatic disease with symptoms in joints or muscles<br>No, I have not been diagnosed with any of these diseases<br>I don't know
**Have you ever been diagnosed by a physician with any of the following other diseases?**	Psoriasis<br>Chronic inflammatory bowel disease, for example Crohn's disease or ulcerative colitis<br>Irritable bowel syndrome<br>Eye inflammation in the form of iritis or acute uveitis<br>Interstitial lung disease<br>Cutaneous lupus<br>Sexually transmitted disease(s)<br>Tension headache<br>No, I have not been diagnosed with any of these diseases<br>I don't know
**Has any of your biological parents or siblings been diagnosed with any of the following rheumatic diseases?**	Rheumatoid arthritis<br>Sjogren's syndrome<br>Systemic lupus erythematosus<br>Myositis<br>Systemic sclerosis<br>Axial spondyloarthritis<br>Osteoarthritis<br>Gout<br>Polymyalgia rheumatica<br>Psoriatic arthritis<br>Fibromyalgia<br>I don't know<br>None of the above
**Has that person been treated by a rheumatologist?**	Yes<br>No<br>I don't know
**Do you smoke?**	Yes<br>No<br>No, but I have been a regular smoker in the past
**How many cigarettes a day do you smoke?**	10 or more<br>1–9<br>I only smoke occasionally
**When did you stop smoking?**	Less than a year ago<br>1–5 years ago<br>More than 5 years ago
**How much alcohol do you consume on average in a typical week?**	An occasional glass<br>Less than 4 glasses<br>4–7 glasses<br>More than 7 glasses<br>I never drink alcohol<br>I don't know

eFigure 1 Flowchart of participant selection

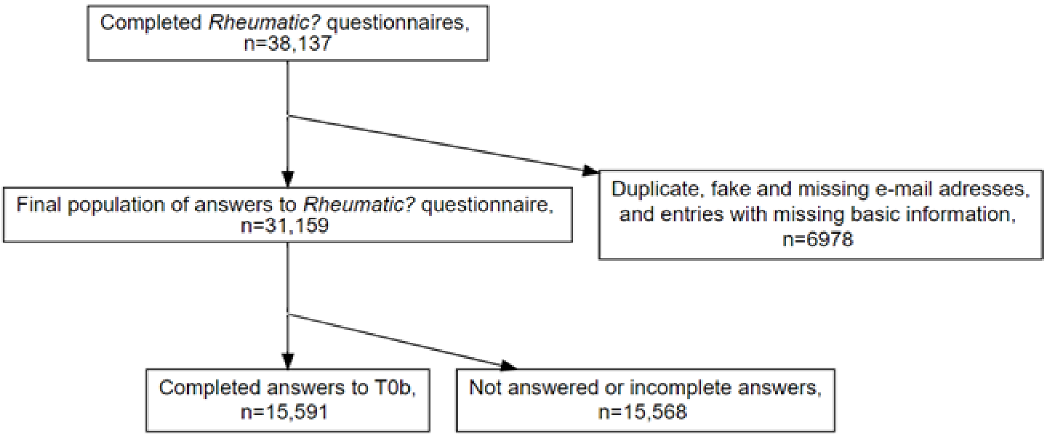

eTable 2 Characteristics of non-responders and responders to the follow-up survey

Characteristic		Non-responders, N = 15,866 (50%)[a]	Responders, N = 15,591 (50%)[a]	Difference in % between responders and non-responders (95 % CI[b])	Overall, N = 31,457[a]
Sex					
	Female	12,139 (77%)	11,844 (76%)	-0.5 (-1.5,0.4)	23,983 (76%)
Age category					
	< 40	4,740 (30%)	1,863 (12%)	-17.9 (-18.8,-17)	6,603 (21%)
	40-50	3,721 (23%)	2,753 (18%)	-5.8 (-6.7,-4.9)	6,474 (21%)
	50-60	4,454 (28%)	5,374 (34%)	6.4 (5.4,7.4)	9,828 (31%)
	60+	2,951 (19%)	5,601 (36%)	17.3 (16.4,18.3)	8,552 (27%)
Smoking status					
	Yes	2,431 (15%)	1,636 (10%)	-4.8 (-5.6,-4.1)	4,067 (13%)
	No	10,307 (65%)	10,414 (67%)	1.8 (0.8,2.9)	20,721 (66%)
	Previous smoker	3,128 (20%)	3,541 (23%)	3 (2.1,3.9)	6,669 (21%)
Alcohol use					
	Never	4,113 (26%)	3,878 (25%)	-1.1 (-2,-0.1)	7,991 (25%)
	Once in a while	5,379 (34%)	5,320 (34%)	0.2 (-0.8,1.3)	10,699 (34%)
	< 4 glasses/week	2,643 (17%)	2,550 (16%)	-0.3 (-1.1,0.5)	5,193 (17%)
	4 - 7 glasses/week	2,380 (15%)	2,522 (16%)	1.2 (0.4,2)	4,902 (16%)
	> 7 glasses/week	1,317 (8.3%)	1,294 (8.3%)	0 (-0.6,0.6)	2,611 (8.3%)
	Don't know	34 (0.2%)	27 (0.2%)	0 (-0.1,0.1)	61 (0.2%)
BMI category					
	<18.5	31 (0.2%)	8 (<0.1%)	-0.1 (-0.2,-0.1)	39 (0.1%)
	18.5-24.9	4,487 (28%)	3,661 (23%)	-4.8 (-5.8,-3.8)	8,148 (26%)
	25-29.9	6,410 (40%)	6,679 (43%)	2.4 (1.3,3.5)	13,089 (42%)
	30+	4,938 (31%)	5,243 (34%)	2.5 (1.5,3.5)	10,181 (32%)
Family diagnosis					
	No	4,518 (28%)	3,837 (25%)	-3.9 (-4.8,-2.9)	8,355 (27%)
	Yes	7,347 (46%)	7,851 (50%)	4 (2.9,5.2)	15,198 (48%)
	Don't know	4,001 (25%)	3,903 (25%)	-0.2 (-1.1,0.8)	7,904 (25%)
Key symptoms					
	Swelling	4,310 (27%)	4,856 (31%)	4 (3,5)	9,166 (29%)
	Pain	12,983 (82%)	13,138 (84%)	2.4 (1.6,3.3)	26,121 (83%)
	Morning Stiffness	9,309 (59%)	10,072 (65%)	5.9 (4.9,7)	19,381 (62%)
	All Day Stiffness	7,388 (47%)	7,662 (49%)	2.6 (1.5,3.7)	15,050 (48%)
	Exhaustion	9,225 (58%)	8,764 (56%)	-1.9 (-3,-0.8)	17,989 (57%)
	Reduced Endurance	6,501 (41%)	6,887 (44%)	3.2 (2.1,4.3)	13,388 (43%)

	None Of The Above	155 (1.0%)	144 (0.9%)	-0.1 (-0.3,0.2)	299 (1.0%)
Previous diagnosis[c]					
	No	9,267 (58%)	6,225 (40%)	-18.5 (-19.6,-17.4)	15,492 (49%)
	Yes	5,145 (32%)	8,465 (54%)	21.9 (20.8,22.9)	13,610 (43%)
	Don't know	1,454 (9.2%)	901 (5.8%)	-3.4 (-4,-2.8)	2,355 (8.1%)

[a]n (%); Median (IQR)

[b] CI = Confidence interval

[c] Self-reported rheumatic-related diagnosis at baseline or in the follow-up survey

eFigure 2 Inclusion frequencies stratified according to recruitment source

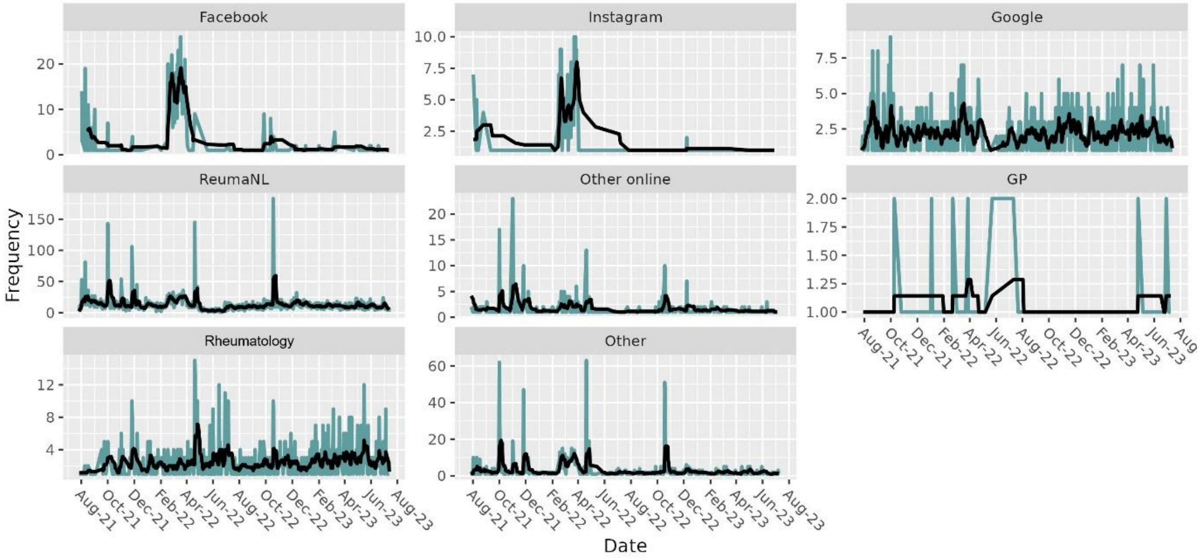

eFigure 3 Website-specific new daily users

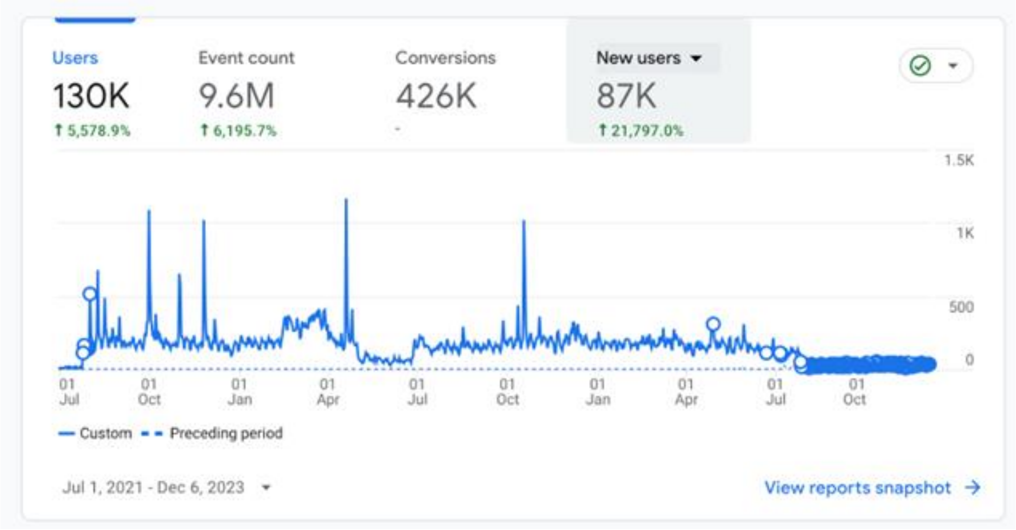

Supplement: Supplementary file 1 [file Data_Sheet_1.PDF]
